# Impact of childhood psoriasis on children and parents during transition to adolescence: An interpretative phenomenological analysis

**DOI:** 10.1111/bjhp.12763

**Published:** 2024-10-29

**Authors:** Marianne Day, Connor Heapy, Paul Norman, Lisa‐Marie Emerson, Ruth Murphy, Olivia Hughes, Andrew R. Thompson

**Affiliations:** ^1^ Department of Psychology The University of Sheffield Sheffield UK; ^2^ School of Health Sciences University of Canterbury Christchurch New Zealand; ^3^ Sheffield Teaching Hospitals NHS Foundation Trust Royal Hallamshire Hospital Sheffield UK; ^4^ School of Psychology Cardiff University Cardiff UK; ^5^ Doctoral Programme in Clinical Psychology Cardiff & Vale University Health Board & School of Psychology, Cardiff University Cardiff UK

**Keywords:** adolescence, childhood, interpretative phenomenological analysis, psoriasis, psychodermatology

## Abstract

**Objectives:**

Psoriasis is a chronic skin condition that can develop at any age. Childhood psoriasis can lead to stigmatization and reduced quality of life in children and parents. This study aimed to gather a detailed family‐level understanding of the experience of childhood psoriasis during the time of transition to adolescence.

**Design:**

A multi‐perspectival interpretative phenomenological analysis (IPA) was used.

**Methods:**

Sixteen semi‐structured interviews with eight parent–child dyads were conducted and analysed in accordance with IPA principles.

**Results:**

Three superordinate themes and three sub‐themes were identified: 1. ‘Transition and transaction’ including: 1.1 ‘Shifting responsibilities and self‐efficacy’; 2. ‘Stigma and social impact’; and 3. ‘The treatment journey’ including: 3.1 ‘Finding an effective treatment’, 3.2 ‘Coping with on‐going management’. Uncertainties surrounding treatment options were an initial focus of difficulty for families. In adolescence, the difficulty shifted to be more identity focussed as the responsibility for disease management and the increased awareness on body image posed added challenges. Both parents and children described visibility and stigma as the most distressing aspects of living with psoriasis and experienced negative emotions that resurfaced during adolescence.

**Conclusions:**

This study suggests that childhood psoriasis can have a significant impact on children, particularly as they begin to transition to adolescence. Findings also highlight the burden of psoriasis for parents. As such, psychological interventions (such as adapted forms of mindfulness‐based Cognitive‐Behavioural‐Therapy) are needed to target and reduce stress. Such interventions are likely to require a systemic focus and support validation of the real impact and fear of stigmatization.

**Statement of contribution**

**What is already known on this subject?**

- Childhood psoriasis is associated with stigmatization, risks of developing mood disturbance and lowered quality of life.
- Childhood psoriasis can affect the wider family and be associated with parental stress.
- Understanding the experience of childhood psoriasis is crucial in informing the delivery of high‐quality care.

**What does this study add?**

- This study uniquely adds an understanding of the nuanced psychological impact of paediatric skin conditions during adolescence.
- The study highlights the need for early management of parental stress including managing cognitions surrounding future fears, with targeted psychological interventions (e.g., mindfulness‐based approaches).
- Young people's concerns tend to be more immediate and shift over time, and assistance may be needed in supporting healthy identity development in adolescence.

## INTRODUCTION

Psoriasis is a chronic inflammatory condition causing red, scaly patches of skin on the body and can also affect the joints (Raychaudhuri et al., 2014). Psoriasis develops in people of all ages, but childhood onset psoriasis occurs in 25%–40% of cases and approximately 2% of children in Europe (Mahé, 2016). Early‐onset psoriasis is often associated with severe physical symptoms and greater psychological impact in later life (Remröd et al., 2013).

Symptoms of psoriasis are often unpredictable, with fluctuations in severity and periods of skin worsening. Although treatments are not curative, good skin clearance can be achieved. Treatments include topical therapies (e.g., corticosteroids, creams), phototherapy, systemic non‐biological therapies (e.g., oral tablets) and biological therapies (e.g., injections) (National Institute for Health and Care Excellence; NICE, 2017). Most children with mild psoriasis are treated with topical therapies, and those with moderate or severe psoriasis are treated with phototherapy and systemic/biological therapies (Seyger et al., 2022). The treatment pathway can involve trying a variety of therapies and finding balance between achieving skin clearance versus avoidance of side effects. For these reasons, the experience of diagnosis, combined with the uncertainties of treatment, can create a significant psychological burden for children and families (Hughes et al., 2022). As such, children with psoriasis have reported a range of negative psychological impacts, including impairment to health‐related quality of life (Randa et al., 2017), stigmatization (De Jager et al., 2011) and depression and anxiety (Kimball et al., 2012).

For children moving into adolescence, the psychological impact of psoriasis could be profound. According to Erikson's (1950) stages of development, adolescence is the time where young people develop their personal identity (i.e., identity versus role confusion) and determines the strength of sense of self across the lifespan. During these years, adolescents experience a range of life transitions resulting in changing social influences, including moving to secondary school, changing friendship groups and gaining independence from the family unit. As well as external factors, the body (e.g., functionality, appearance) and inner experiences (e.g., emotions) play an important role in identity formation. An adolescent with psoriasis may experience a disruption during this stage of development, which could impact emerging self‐esteem (Fox et al., 2007).

Indeed, adolescents with psoriasis can experience issues with appearance, self‐esteem, anxiety, loneliness and a lack of emotional and social support (Fox et al., 2007; Rasmussen et al., 2018). While for parents of children with psoriasis, there can be negative impacts on health and well‐being, family life and work/social functioning (Rasmussen et al., 2020; Tollefson et al., 2017). Despite this, less is known about the experiences of families, as existing research has tended to focus on either the child or parent, and could overlook important information. For example, Rasmussen et al. (2018, 2020) interviewed children and parents and highlighted differences in perspectives on adherence to treatment and the psychosocial impact of psoriasis. However, interviews were conducted and reported separately with a sub‐sample of parents, and the children were older adolescents/young adults. Extending this approach to simultaneously investigate the different perspectives of parents and younger children/adolescents and analyse perspectives within dyads may provide further insights into how families manage psoriasis. Gathering data on family functioning could support the identification of psychological variables to target with interventions (Ablett & Thompson, 2016).

Phenomenological forms of qualitative research are useful for investigating lived experiences and could contribute to a new theoretical understanding of how families affected by skin conditions adjust (Larkin & Thompson, 2012). Therefore, the current study used a qualitative design to interview children with psoriasis and their parents. Interpretative phenomenological analysis (IPA: Larkin et al., 2006; Smith et al., 2009) has been previously used to develop nuanced accounts of health conditions, including those affecting appearance (e.g., Moss et al., 2020; Rafique & Hunt, 2015; Thompson et al., 2002; Thompson & Broom, 2009). The aims of the present study are to (a) investigate children's experiences of psoriasis; (b) investigate parental experiences of childhood psoriasis; and (c) determine the impact of psoriasis on parent and child psychological well‐being and family functioning, in relation to the period of transition to adolescence.

## MATERIALS AND METHODS

### Participants

Ethical approval was obtained from the United Kingdom's NHS Ethics Committee (Ref: 18/YH/0134:237241). Data collection for the study took place in 2018–2019 from across the United Kingdom. Parent–child dyads were recruited via social media (two families) and via NHS dermatology clinics (six families). Parents provided informed consent for themselves, and their children aged under 18 years to take part; children also gave assent. This sample size was sufficient for meaningful idiographic analysis (Larkin & Thompson, 2012; Thompson et al., 2011). To be included in this study, children were required to be aged between 10 and 16 years (see Table 1), have a diagnosis of psoriasis, and be receiving treatment. Parents needed to identify as a significant carer of the child.

**TABLE 1:** Participant demographics (*n* = 8 parent–child dyads).

Parent demographics (*n* = 8)	Number	Child demographics (*n* = 8)	Number
Age range (in years)	8	Age range (in years)	8
33–49		11–16	
Family ethnic group		Age at diagnosis (in years)	
White British	6	2–11	
British Asian‐Pakistani	1		
Black African British	1		
Parent severity ratings for child's psoriasis		Child severity ratings for child's psoriasis	
Mild	2	Mild	2
Moderate	4	Moderate	5
Severe	2	Severe	1
Parent educational level		Family relationship with psoriasis	
Secondary	1	Parent	4
Post‐secondary (A‐level equivalent)	3	Sibling	2
Undergraduate university degree	1	Grandparent	3
Postgraduate university degree	3	Aunt/uncle	2
FDLQI scores: impact of Condition 0–30 (% out of total score of 30)		None	1
1 (3.33%)	1	Other medical conditions	
3 (10.00%)	1	Asthma	1
7 (23.33%)	1	Joint problems (e.g., psoriatic arthritis)	1
8 (26.66%)	1	None	6
13 (43.33%)	1	CDLQI scores: effect on child's life (0–30)	
14 (46.66%)	1	No effect on child's life (0–1)	0
19 (63.33%)	1	Small effect (2–6)	4
20 (66.66%)	1	Moderate effect (7–12)	4
		Very large effect (13–18)	0
		Extremely large effect (19–30)	0

*Note*: Score range: 0–30. Higher scores reflect greater QoL impairment.

Abbreviations: CDLQI, Children's Dermatology Life Quality Index; FDLQI, Family Dermatology Life Quality Index.

### Measures

Demographic information was collected to add additional contextual information (see Table 1). Participants completed paper measures prior to taking part in qualitative interviews, and children completed questionnaires separately.

Parent QoL was assessed using the Family Dermatology Life Quality Index (FDLQI) (Basra et al., 2007; Basra & Finlay, 2007). The FDLQI measures the impact of a skin condition on QoL with 10 items rated on a 4‐point Likert scale (e.g., ‘Over the last month how much emotional distress have you experienced due to your relative/partner's skin disease [e.g. worry, depression, embarrassment, frustration]?’) (Basra et al., 2007). Child QoL was assessed using the Children's Dermatology Life Quality Index (CDLQI: Lewis‐Jones & Finlay, 1995). The CDLQI measures the impact of a skin condition on a child (aged 4–16 years) QoL with 10 item rated on a 4‐point Likert scale (e.g., ‘Over the last week, how embarrassed or self‐conscious, upset or sad have you been because of your skin?’; (Lewis‐Jones & Finlay, 1995).

### Interviews

Interviews were conducted following guidance for data collection of sensitive topics (Thompson & Russo, 2012). Semi‐structured interviews were held in the University (one family) or at the family's home (seven families). Questions were informed by previous literature, existing QoL questionnaires (e.g., CDLQI/FDLQI) discussion with an expert‐by‐experience (i.e., an adolescent with psoriasis) and discussions between members of the research team (including a dermatologist who regularly works with families affected by psoriasis). To uphold ethical practices with consideration of the possibility that children may have felt pressure from their parents to participate, we interviewed children separately and regularly checked they were happy to continue.

Parents were asked about the psychosocial impact of psoriasis on their child, and children were asked about their experience of living with psoriasis (see Appendix S1). The interview schedule was piloted with a child with psoriasis and their parent to ensure questions were comprehensive. At this stage, only minor changes were required to aid understanding. Parental interviews lasted 25–44 min (mean = 33 min). Child interviews lasted 15–58 min (mean = 36 min). Interviews were recorded and transcribed verbatim. Data were anonymized and pseudonyms used to protect identities. Families were given £25 as compensation for their time.

### Data analysis

Data analysis was carried out in accordance with IPA principles, using a four‐stage process (Smith et al., 2009). Transcripts were read to gain familiarity with the issues raised. Annotations were made in the margins of each transcript to note points of interest. Tentative themes were identified for each interview and then analysed as a pair to consider relational issues. Each transcript was read again to develop interpretation of initial themes and draw out subjective meanings for individual families. Themes were then compared across dyads to identify similarities/differences. Connections between themes were mapped to develop a structure of master themes/sub‐themes. Finally, interviews were re‐read using this thematic structure to ensure it was inclusive.

### Quality control and reflexivity

The integrity of methodology was determined by frequent cross‐checking, following established guidelines (see JARS‐Qual; APA, 2020; Levitt et al., 2018). Each stage of data analysis was verified by another member of the research team with experience of IPA, using an audit template developed specifically for this purpose. A reflexivity notebook was used throughout the analysis to highlight the origin and revision of themes (Biggerstaff & Thompson, 2008). All authors reviewed and agreed the final thematic structure.

The interviewer (C.H.) was a White, British male who received training from a senior clinical health psychologist (A.R.T.) with extensive experience in qualitative interviewing and providing support to dermatology patients. The interviewer had lived experience of another visible skin condition (acne) that developed during adolescence. While this experience helped the interviewer empathize with participants in this study, there was a risk of leading questions that fit his own personal experience. Regular reflection and discussion within the full research team helped reduce this risk and ensured that each individual's experience was adequately captured.

## RESULTS

Three superordinate themes and three sub‐themes were identified (see Table 2) and will be discussed with supporting quotes from participant interviews.

**TABLE 2:** Summary of themes and sub‐themes.

Theme	Sub‐theme
1. Transition and transaction	1.1 Shifting responsibilities and self‐efficacy
2. Stigma and social impact	
3. The treatment journey	3.1 The search for an effective treatment
	3.2 Control, not cure—on‐going management

### ‘Transition and transaction’

This theme encompasses the relationship between the parent and child (transaction) in relation to the impact of psoriasis and the child's developmental stage (transition to adolescence). Parents described the impact psoriasis had on family life. Routines had changed to revolve around treatment, and there were also financial/social implications. Parents and children described the time burden of applying creams. Parents sometimes reframed this as an opportunity for bonding, while children only reported finding it tedious and uncomfortable. Lucy (young person) described how the application of cream caused annoyance between her and her mother: ‘I don't think she notices, but it's like a passive aggressive sigh, when her arms ache or her neck aches, and I'm sat there like “my neck is dead”’. Zoe (parent) felt guilty applying creams when Joshua (young person) was upset: ‘He's saying, “I hate this, I wish I didn't have this”, which is obviously upsetting’.

Applying cream became more contentious as children got older and was relevant to the developmental stage of this sample progressing into early adolescence. As children were beginning to strive for independence, they experienced embarrassment from invasions of privacy during treatment. For parents relinquishing control, there was a balance of attempting to remind their children about adherence to treatment without overshadowing their interactions:

…I will spot something has gone dry – “have you creamed it? How many times have you creamed today?” … I'm conscious that it's a lot of our conversation. And I only just manage to hold back sometimes. (Charlotte; Parent)

Parents expressed a sense of ‘loss’ in relation to these changes to ‘normal’ family life/relationships. Mary described how psoriasis had consumed their ‘family life pattern’ until they had become used to new routines. Ayesha (parent) felt she had missed out on early bonding experiences with her daughter, Saba (young person), as her psoriasis had meant she could not be physically held. In the longer term, Ayesha (parent) and Saba's (young person) relationship had been affected by Saba's psoriasis‐related anger issues. Ayesha (parent) felt that their relationship had been defined by psoriasis:

You're meant to enjoy it and you're meant to show your baby off…I've not known a time when things were not about her skin. (Ayesha; Parent)

Psoriasis impacted parental well‐being and precipitated anxiety. Initially, parents felt powerless, desperate and frustrated as they could not do anything to improve their child's condition. These feelings often returned when symptoms worsened:

You feel powerless … as a parent you just want to make things better for your child and you can't … you're just waiting, hoping that his skin will improve. (Abigail; Parent)

Parents worried their child's self‐esteem was being damaged by psoriasis and observed their children missing out on activities and becoming socially withdrawn. Abigail (Parent) thought Alex (young person) was ‘not living the normal life of a 14‐year old boy’, while Martin (parent) worried about Isaac's (young person) confidence: ‘I wonder what he'd be like if he never had any of that’. Worry was projected into the future and parents worried their children could be bullied in secondary school or that educational opportunities and romantic relationships would be negatively affected. To reduce worry, parents tried to focus on the present and ‘just get on with it’, and:

…Not to think “what might happen?” “Might she end up hospital?” “Might things get worse?” Learning to think “that hasn't happened today, that didn't happen yesterday and all the days before”… (Charlotte; Parent)

Children's worries were more immediate and surrounded issues such as settling into a new school, changing clothes for physical education (P.E.) or whether people would stare on holiday. Only Mia (young person), who was 16 years old, said she worried about the impact of psoriasis when she was older (in romantic relationships). Like their parents, child anxiety could be overwhelming. Lucy (young person) described how her psoriasis played on her mind and how she wanted to ‘feel free’ of it. Children also recognized discrepancies between their life and how it was ‘supposed’ to be (James; Young person). Young people compared themselves with friends and siblings, wanting to be like others (Saba, Lucy, Alex) or wishing for *‘nice skin’* (Isaac; Young person).

Parents were concerned that children would pick up on their anxieties and avoided talking about sensitive subjects. Ella (parent) shared Rachel's (young person) sadness at the hair loss caused by scalp psoriasis and avoided the subject. Other parents did not want to ‘trigger’ anxiety (Zoe; Parent) or ‘push it’ (Abigail; Parent) by asking questions. Charlotte's (parent) attempts to reduce Lucy's (young person) anxiety by saying her psoriasis ‘will go very soon’ had a negative impact:

She's trying to make me feel better but it's not exactly working … it makes me quite upset. (Lucy; young person)

Children found it embarrassing to talk to parents and friends about their psoriasis. This sense of shame affected relationships, adjustment into new schools, and made it difficult for parents to evaluate the impact of psoriasis and address anxieties. However, communication difficulties were not universal. Having another family member with psoriasis increased empathy and was a source of support and information for children. Despite this, some parents expressed guilt around a family history of psoriasis. As Susan (parent) said, ‘you can feel it's your fault’.

### Shifting responsibilities and self‐efficacy

In five families, the child had taken over some (or all) of the responsibility for treatment. This was characterized in all cases by a period of less well‐managed skin and some parent–child tension over the shift in responsibility. Parents supported their child's independence but worried their child was not applying cream properly:

I'm seeing it less often now; when I have seen it, ‘bah it's getting worse, please can I take it over again and do a bit more? (Charlotte; Parent)

Children agreed they did not apply cream as strictly as their parents, and their approach was more reactive than preventative. Children also admitted they needed to be reminded to do treatments, but none felt their parents were nagging them and found the encouragement reassuring. Some children had stopped treatment for a short period to see ‘what would happen’ (Mia; Young person) or avoided treatments which were visible to others. Mia (young person) described the burden of regular cream application:

To constantly have to be putting [cream] on, you just think, “oh, I just want a break from it”. But if I do, then it gets worse. (Mia; Young person)

In line with the child's developmental age, parents took a longer‐term view of treatment and described how their children were prone to de‐motivation when their efforts at self‐management did not result in a ‘cure’:

[Lucy] (Young person) didn't want to keep [applying treatment] because she was discouraged that it didn't matter if she did … it didn't make it go away. (Charlotte; Parent)

The association between perceived self‐efficacy and the child's outcomes was highlighted. Isaac (young person) described how he felt ‘really proud…because I've come all this way and it's getting better’.

### Stigma and social impact

This theme describes relationships with other people which centred on the family's experiences of psoriasis. The psychological impact of a visible skin condition was greater than the impact of physical symptoms for children and parents. Visibility included the presentation of skin lesions (e.g., redness or dryness), the traces left behind (e.g., flakes of skin or blood) and the effects of treatment (e.g., greasy hair and shiny skin). All of the children felt self‐conscious in public and covered their psoriasis whenever possible. Situations such as swimming and getting changed in front of peers for P.E. at school were situational triggers for anxiety. Anxiety was greatest for children and parents when the condition affected areas which could not be covered, particularly the face and scalp. The experience of anxiety was profound for older children, who appeared to be more conscious of their appearance:

It's a private condition when it's not on his face … it becomes a very public one and then that's where he gets anxious, and we worry … (Martin; Parent)

Children and parents recounted situations where other people had demonstrated shock or disgust. Children were asked: ‘what's that gross stuff on your skin?’ (Lucy; Young person), ‘why you so red?’ (Saba; Young person) or ‘what have you done to yourself’ (Mia; Young person). Lucy (young person) had noticed people backing away which ‘doesn't make you feel very nice’. All families said their child had experienced bullying and name calling, often related to fears of contagion (e.g., ‘rash’, ‘disease’, ‘lurgy’, ‘chicken pox’). These negative social encounters led children and parents to anticipate future negative reactions. Alex (young person) described how memories of being bullied led to anger issues: ‘I used to get flashbacks a lot and then people asking all the time, it built up’, while Lucy (young person) worried that people were ‘looking at me funny. It's sort of in my consciousness most of the time’. Some children also said that other people's responses to their skin had not been as bad as they had expected. For example, Alex (young person) had initially socially withdrawn during a holiday with other families, but later found no one commented on his psoriasis, ‘I ended up taking both my jacket … I put shorts on as well … and I was fine, no one said ‘owt’.

The burden of unwanted attention and having to explain psoriasis to other people was the main reason for children covering up. Parents and children attempted to avoid questions by providing information before they were asked. Parents gave teachers information about psoriasis to share at school. Martin (parent) described how Isaac (young person) tells people about his psoriasis the first time he meets them, ‘he just likes to declare it … get it out of the way so he's not worried’. Explaining psoriasis was more difficult as people had usually not heard of it. Confusion with eczema contributed to feeling misunderstood and to being given ‘lectures’ (Saba; Young person) or ineffective ‘cures’ (Parents: Charlotte, Martin). Zoe (parent) wondered whether eczema is ‘more acceptable’. Parents also worried that their child would feel different or isolated because psoriasis was not well‐understood. Martin (parent) did not want Isaac (young person) to feel like an ‘outsider’. Negative appraisals of psoriasis were not only experienced through the reactions of other people, and some children expressed self‐disgust:

I'll be wearing a short‐sleeved t‐shirt … and I'll be looking at my arms, because I've got psoriasis on my arms and be like ‘eugh’. (Lucy; Young person)

Parents expressed sadness and discomfort with their child's appearance. Martin (parent) admitted feeling embarrassed in public when Isaac's (young person) psoriasis was visible. Ella (parent) said she would not feel comfortable going outside if her hair looked like Rachel's (young person), and Saba (young person) found Ayesha's (parent) appearance difficult as a baby:

When she was really young and covered in these creams, just the sight of her… obviously, she was my child and I loved her, but it wasn't nice watching her, because she didn't look like the normal child. (Saba; Parent)

Some parents also felt that they were being judged. Ella (parent) worried that people would think she was not washing Rachel's (young person) hair or treating her skin. Charlotte (parent) presumed a doctor thought she was an *‘incompetent mother’* and Ayesha (parent) believed ‘people think she's done something wrong … for her to have it’. She described an occasion in hospital when Saba's (young person) symptoms had been misinterpreted by a student doctor as burns:

…she's not been burnt…but somebody in the medical field who is trained and educated, the visual signs, looked like she'd been dipped in hot oil. (Ayesha; Parent)

### The treatment journey

This theme describes how families progressed through a series of treatment stages: from the initial search to on‐going management and shifting responsibilities of care as the child got older. Effective treatment was a vital part of how families coped. However, accepting permanency was something families had to come to terms with.

### The search for an effective treatment

Parents described the time and effort involved in finding an effective treatment. While children sometimes recalled these stages, they were not aware of the impact it had on their parents. In most cases, psoriasis started with mild symptoms and children and parents expected it to be cured quickly. As psoriasis progressed, feelings of anxiety increased:

The patch under the eye … we were like “oh it's just a thing, it will go” and no one cares what you look like at three … but when it turned into a body thing … I was really worrying about what it was and how long it was going to be there … (Martin; Parent)

Cycles of emotion began with hope, followed by feeling ‘shattered…like another smack in the face’ (Ayesha; Parent) if a treatment was unsuccessful. As Martin (parent) described; ‘any new treatment, you've got to really invest in it to see any kind of payoff … it's a gamble’. Many treatments had adverse effects which had to be balanced against the level of physical/psychological debilitation of psoriasis. Sometimes treatments were judged as ‘not worth it’ (Susan; Parent), and other times they were ‘a small price to pay’ (Ayesha; Parent). During the initial period of treatment, parents sought out alternative treatments and attempted to identify triggers. Ayesha (parent), said ‘anything that we were suggested, we gave it a shot’.

The reassurance of finding an effective treatment offset the uncertainty of the future. However, this sense of security was fragile. The possibility that treatment might stop working meant parents could feel stressed, even when their child's present treatment was effective. Ayesha (parent) described her daughter's treatment as ‘the only thing keeping her afloat’, while Michelle (parent) thought, during a period of well‐managed skin, ‘another month down the line, it is going to be back to square? Which I think is psoriasis…’.

### Control, not cure – on‐going management

Parents and children talked about the disappointment when symptoms recurred. Families had been told that psoriasis could be lifelong, but many still hoped there could be a cure. Ella (parent) wanted to ‘go back to how it was’, while Martin (parent) hoped ‘we can forget about it and we can move on’. Both Abigail (Parent) and Saba (young person) wanted to ‘wave a magic wand’ to make it better. In most families, the child's skin had improved, but there was frustration that psoriasis had not cleared completely. Martin, Michelle and Ayesha (parents) talked about periods of flare:

We knew that meant that it was going to be a new round of it…even though it doesn't look that bad, you're seeing it, thinking… ‘this is going to be another 6‐month journey’. (Martin; Parent)

Over time, families accepted that management of psoriasis would be ongoing with the aim of achieving well‐managed skin, rather than a cure. Families talked about dealing with symptoms as they occurred:

I've been told it will come and go throughout my life, and I can have a few years without it affecting me and then it could come back. So … when it's here, got to treat it … it's just about keeping on top of it. (Mia; Young person)

Most families were resigned to the fluctuations in severity but talked about ‘being stuck with it’ (Martin; Parent). Two families had not accepted the non‐curable nature of psoriasis:

…They said she might grow out of it and we're praying that she does, because if she doesn't, I don't know what will happen. (Ella; Parent)

## DISCUSSION

This study investigated the impact of psoriasis on children and their parents. Families identified sources of stress, including negative social reactions to the child's psoriasis, transactional effects on the parent–child relationship, uncertainty and the lack of control inherent to managing a chronic condition. These findings are novel and importantly contribute to the gap in literature surrounding the experience of childhood psoriasis during the period of transition to adolescence. As well as this, parental accounts have shed light on the wide‐ranging consequences of childhood psoriasis for the child's parents and functioning of the family unit (Hughes et al., 2022).

Psoriasis impacted family communication and relationships. There were implications in terms of shifting responsibilities for psoriasis management during adolescence as children began to take control over their own body. Families described the difficulty of negotiating responsibilities in the context of parental monitoring and limited communication. Children did not always manage their skin effectively and parents struggled to relinquish control. In these cases, children became discouraged with self‐management when their efforts did not lead to a ‘cure’. For other families, children had developed anger management issues. This heightened state of emotion had consequences for relationships and could increase potential for family conflict. These findings demonstrate the need for parents to encourage realistic expectations and to be sensitive to child self‐blame when symptoms reoccur.

The dyadic perspective on the challenges of treatment was highlighted, as many parents described feeling frustration at the non‐curable nature of their child's psoriasis. Acceptance of permanency and having an established treatment were important factors in parental stress levels (Rasmussen et al., 2020). Here, families who had accepted non‐cure had gone through a number of periods of skin worsening and learned to focus on dealing with symptoms as they arose. Focusing on symptoms (rather than a cure) has been identified as an effective coping strategy in other paediatric chronic illnesses, while avoidant coping (e.g., denial, wishful thinking) is consistently associated with the poorest outcomes in childhood and adolescence (Compas et al., 2012). Indeed, the treatment‐related stresses described by families are supportive of previous literature (Rasmussen et al., 2020). Parents struggled with feeling powerless as they progressed through the treatment pathway. Treatments from health‐care professionals did not always work, and there were often adverse side effects which had to be weighed against benefits. However, improvements in the child's skin and well‐being were seen as worth it for the extra risks.

Although some families had given up certain treatments or reported negative experiences with health‐care professionals, none had withdrawn from care. All families sought an effective treatment and were reliant on clinicians. These findings surrounding interactions with health‐care professionals differ from Rasmussen et al. (2020), where parents felt uninformed, lacked trust and avoided treatments to protect their child from the short‐ and long‐term effects. Ineffective treatments, adverse effects and recurring symptoms have also been found to reduce adherence to treatment for children with other skin conditions (Smith et al., 2010, 2013). Therefore, it is important to consider the potential impact of these factors on parental management of skin conditions, including the ‘handover’ of managing treatment that occurs as children approach adolescence and are brought into shared decision making with health‐care professionals.

Psoriasis caused considerable parental stress and impacted well‐being. Parents experienced disruptions to normal life (changed family relationships, time pressures, adapted family activities). Some children could not be physically handled, which was particularly difficult for parents when they were babies. Parents also described situations where they were unable to physically comfort their child or had to deal with anxiety and resistance around treatment. However, although families described applying creams as an opportunity for bonding (Ablett & Thompson, 2016), it could also be a source of frustration, boredom and tension. Both parents and children expressed discrepancies between how they perceived their life and how they thought it was ‘supposed’ to be. For parents, there was a loss of ‘normality’ as psoriasis had led to changed expectancies of family life, with feelings of guilt, sadness and worry. Although the study results are in line with previous findings, there are also some differences. For example, this sample did not report acute treatment‐related problems in parents (e.g., sleep problems, personal care, loss of career, marital discord) (see Tollefson et al., 2017). It is possible that children in this study were further into treatment and the immediate burden of care had reduced. Indeed, Ablett and Thompson (2016) found that the greatest impact on parents was physical care in children under the age of 5 years.

Children were more impacted by having a visible skin condition and felt shame and unfairness from social comparisons. These feelings can be explained using self‐discrepancy theory (Higgins, 1987), which highlights the emotional discomfort caused by mismatches between the actual and ideal/ought self. For example, beauty stereotypes advertised in the media could be internalized and lead to body dissatisfaction from the unsuccessful attempt to attain a flawless appearance (Tiggemann & McGill, 2004; Trekels & Eggermont, 2017). Indeed, Carter and Vartanian (2022) reported that people with lower self‐concept clarity (Campbell, 1990) might be more likely to carry out comparisons to idealized images. The role of comparison could be important when considering the vulnerabilities of adolescent years for appearance‐related concerns and the development of identity as the impact of visible psoriasis could result in negative effects for self‐esteem lasting into adulthood (Fox et al., 2007).

In an attempt to buffer concerns, it has been previously reported how parents sought to ‘normalize’ their child's psoriasis by downplaying its significance and were later surprised by the psychosocial impact it had (Rasmussen et al., 2020). However, in the present study, parents had long‐been aware of the impact of psoriasis on their child's confidence and self‐esteem from a young age and were even anticipating the effects on later life. Both children and parents described experiencing distress from the responses of other people to the child's skin. The potential for negative judgement had led to children experiencing the desire to cover up and highlighted the burden of disclosure. The impact of negative evaluations extended to the anticipation of judgement from other people. For children, there could be a predisposition to an increased fear of social evaluations associated with the normative pattern of development during adolescence (Westenberg et al., 2007). In this study, negative social reactions had gone beyond the situations in which they occurred, to have a long‐term impact on child self‐esteem, child and parent anxiety, and child behaviour. For example, negative social experiences (perceived and anticipated) led to shame and embarrassment and meant that children (and sometimes parents) withdrew socially, missed out on activities and felt unable to talk to others.

The potential for reduction in social support experienced by children may create a vicious cycle, and social isolation could further exacerbate the burden of psoriasis or even precipitate avoidance (Barlow et al., 2023). Other studies have also demonstrated stigma associated with having a visible skin condition (e.g., Fox et al., 2007; Rasmussen et al., 2018) and the association between negative peer reactions, appearance dissatisfaction and depressive symptoms (Barlow et al., 2023; Feragen & Borge, 2010; Feragen & Stock, 2016). Moreover, the impact of negative evaluations had potential to extend to influence parenting behaviour as some parents also felt stigmatized and worried that other people might think the child's condition was their fault.

A number of limitations should be considered when interpreting these findings. The families who agreed to participate in the study may not be representative of other families of children with psoriasis. The families were also predominantly white British and only one interview included a father. Future research should employ purposive sampling to ensure that participants from a range of ethnic backgrounds, as well as fathers, are recruited. It is also possible that children avoided talking about some aspects of their experience during the interviews as embarrassment was a pervasive feature and might explain why children did not mention certain experiences that were evident in parent interviews.

However, our study also has several strengths. Although there is a large body of research illustrating the significant psychological burden of living with a skin condition, less is known about the impact on parents. Furthermore, by recruiting parent–child dyads, it was possible to contrast parent and child experiences of psoriasis. Importantly, the findings suggest that skin‐related distress can extend to affect the entire family unit, and this has important implications for assessment of intervention or/and support.

## IMPLICATIONS

Much of the literature on chronic skin conditions has focused on the considerable physical and psychological burdens, but there is a need to consider how families cope (Ablett & Thompson, 2016). Our study highlights the importance of offering tailored psychological support to children diagnosed with skin conditions, including psoriasis, and their parents at an early stage in their care. Indeed, the British Society for Paediatric and Adolescent Dermatology has recently drawn upon expert opinion to recommend the use of specific patient‐rated mental health outcome and quality of life measures, such as the Revised Children's Anxiety and Depression Scale (RCADS) (Chorpita et al., 2000 [see McPherson et al., 2023 for full recommendations]). Further, in order to embed psychosocial assessment into dermatology service delivery, McPherson et al. (2023) have developed a history taking aid (‘You and Your Skin’). Clearly, the findings of our study further demonstrate the need for additional psychological training and support to be provided to core dermatology staff.

As described in this study, positive coping strategies included family support and communication, sharing information with others and dealing with psoriasis in the present rather than worrying about the future. Parenting interventions for children with chronic illnesses have focused on disease management, positive parenting and family communication (Morawska et al., 2015). Specifically, families of children with psoriasis may also require support to manage anxiety around the child's future, acceptance of non‐cure and to manage and reframe negative social interactions to avoid anticipatory anxiety and social withdrawal (Ablett & Thompson, 2016). The families in this study often referred to dealing with psoriasis in the present, accepting uncertainty and not dwelling on worries about the future.

Therefore, mindfulness‐based interventions focusing on present attention and responsiveness may improve outcomes for families of children with psoriasis and assist with managing judgement and negative reactions from other people. ‘Mindful Parenting’ (Kabat‐Zinn & Kabat‐Zinn, 1997) may help parents to deal with difficult parenting situations in the context of psoriasis, such as creaming and negotiating shifting responsibilities for management and are likely to have benefits for the parent–child relationship. This may improve family communication, reduce stress, improve management of the child's skin and improve child adjustment (Bögels & Emerson, 2019; Emerson & Bögels, 2017). Interventions designed to increase mindful parenting could be beneficial for parental distress (e.g., experiential avoidance) and have subsequent positive outcomes for the child via parenting behaviours (Emerson et al., 2019). The approach has shown promise for behavioural conditions (e.g., ADHD/ASD: Singh et al., 2010; van der Oord et al., 2012), chronic illness (Serkel‐Schrama et al., 2016), mental health (Bögels et al., 2014) and more recently, for reducing parental stress in parents of children with psoriasis and eczema (Heapy et al., 2022).

## CONCLUSION

This study investigated the perspectives of children with psoriasis and their parents during a time of transition into adolescence and identified the shifting burden of care. An initial focus on finding an effective treatment and physical care gave way to disappointments at the fluctuating nature of psoriasis symptoms and anxiety about the long‐term consequences for the child. Unlike previous studies, parents were equally as focused on the psychosocial impact of psoriasis as their children. Children and parents highlighted the pervasive impact of visibility and stigma for both groups, but also how these were experienced differently. Future interventions should promote positive management in the context of adverse effects, the uncertainty of treatments and symptoms, and also address stigma, anticipatory anxiety, coping with non‐cure and shifting responsibilities during adolescence.

## AUTHOR CONTRIBUTIONS

**Marianne Day:** Writing – original draft; writing – review and editing; methodology; formal analysis; investigation. **Connor Heapy:** Investigation; writing – review and editing; project administration; conceptualization; methodology. **Paul Norman:** Investigation; writing – review and editing; funding acquisition; conceptualization; methodology. **Lisa‐Marie Emerson:** Investigation; writing – review and editing; funding acquisition; conceptualization; methodology. **Ruth Murphy:** Investigation; writing – review and editing; funding acquisition; conceptualization; methodology. **Olivia Hughes:** Investigation; writing – review and editing; methodology. **Andrew R. Thompson:** Investigation; writing – review and editing; writing – original draft; funding acquisition; conceptualization; methodology; formal analysis; project administration.

## FUNDING INFORMATION

The study was funded by a grant from the Psoriasis and Psoriatic Arthritis Alliance (PAPAA) (grant number: 1522 24).

## CONFLICT OF INTEREST STATEMENT

None to declare.

## ETHICS STATEMENT

Ethical approval was granted by the United Kingdom's NHS ethics committee (reference: 18/YH/0134:237241).

## Supporting information


Appendix S1

## Data Availability

The data that support the findings of this study are available from the corresponding author upon reasonable request.
