# Supplementary material for: Impact of childhood psoriasis on children and parents during transition to adolescence: An interpretative phenomenological analysis
Source: Br J Health Psychol. 2024 Oct 29;30(1):e12763. doi: 10.1111/bjhp.12763 (PMC11586832; doi:10.1111/bjhp.12763)
Supplement: Supplementary file 1 — Appendix S1 [file BJHP-30-0-s001.docx]

**Impact of childhood psoriasis on children and parents during transition to adolescence: An interpretative phenomenological analysis**

(Supplemental Files)

Marianne Day,^1^Connor Heapy,^1^ Paul Norman,^1^ Lisa-Marie Emerson,^2^ Ruth Murphy,^3^ Olivia Hughes^4^ and Andrew R. Thompson.^1,5*^

^1^ Department of Psychology, The University of Sheffield, Cathedral Court, Sheffield, S1 2LT

^2^ School of Health Sciences, University of Canterbury, Christchurch, New Zealand.

^3^ Sheffield Teaching Hospitals NHS Foundation Trust, Royal Hallamshire Hospital, Glossop Road, Sheffield, S10 2JF.

^4^ School of Psychology, Cardiff University, Tower Building, 70 Park Place, Cardiff, CF10 3AT.

^5^ Doctoral Programme in Clinical Psychology, Cardiff & Vale University Health Board & School of Psychology, Cardiff University, 11th Floor, Tower Building, 70 Park Place, Cardiff, CF10 3AT.

*Corresponding author’s present address: Professor A. R. Thompson, Programme Director, Doctoral Programme in Clinical Psychology, Cardiff University, 11th Floor, Tower Building, 70 Park Place, Cardiff, CF10 3AT, thompsona18@cardiff.ac.uk, +44(0)2920870582.

**Child interview schedule**

*Preamble:* Okay, so thank-you for filling out those questionnaires for me. So now I’d just like to spend some time talking to you about your experience of psoriasis. Some people find it harder than others to talk about this this type of thing, so it’s okay to tell me as much as you feel comfortable talking about. Also, don’t worry if you feel like you are doing all the talking! I’ve brought along some drawing materials, because sometimes it’s easier to draw things, than say how you feel out loud. You don’t have to use these though. I’ll let you decide what feels more comfortable as we start talking. Do you have any questions before we start again? *(Switch tape recorder on)*

1. **Can you tell me a little bit about yourself “name of child?”**

Prompts: What do you do for fun? Have you got any brothers or sisters? Can you tell me a little about your friends?

1. **Can you tell me when you first started noticing problems with your skin?** *(Check whether child uses the term psoriasis)* **OR I wonder if you could draw a quick picture of yourself for me?**

General Prompts: What happened? How did you feel? What were you thinking?

Picture Prompts: Can you tell me about your picture? (specific/unclear parts: What is this part? Can you explain what is happening here? What does this bit mean? Can you tell me some more about your picture? What are you thinking/feeling here?)

**3. Can you tell me a bit about school and hobbies? If it’s easier to draw you can?**

Prompts: What is school like? What is it like meeting new people? How do you feel about your friends? How do you think other people feel/think about you?

Picture Prompts: As above

**4. How has life been since you noticed changes in your skin? If it’s easier to draw you can?**

Prompts: Has your psoriasis changed anything? How have other people been? What have your teachers/friends/people in the family been? How does that make you feel? What do you think about that?

Picture prompts: As above

**5. How have things been in your family since you have had the changes in your skin (use child’s label for the skin condition)?**

Prompts: Has your psoriasis changed anything in your family? How have other people in your family been about your skin condition (go through each i.e. mum, dad, siblings)? Prompt – what sorts of things have been said/done in connection with your skin condition

**Adult interview schedule**

*Preamble:* Okay, so thank-you for completing the questionnaires. As I said earlier, this conversation is for me to find out a little more about your experience of “name of child’s” psoriasis, so don’t worry if you feel like you are doing all the talking. If you would like to stop at any point, or do not feel able to answer a question please let me know. Do you have any questions before we start?

(*Switch tape recorder on)*

**1. When did “name of child’s” psoriasis start? Can you tell me about this time?**

Prompts: What happened? How did you feel? What were you thinking?

**2. Can you tell me about what it’s like being “name of child’s” mum/dad/ ‘care-giver’s relationship with child’?**

Prompts: Has ‘name of child’s’ psoriasis change things in any way? How do you feel/think about those things? What do you think your child feels/thinks about them?

**3. Can you tell me about ‘name of child’s school life?’**

Prompts: Has ‘name of child’s’ psoriasis changed things in any way? How do you feel/think about that?

**4. How has life been since ‘name of child’s’ psoriasis?**

Prompts: What have you found helpful/unhelpful? What have the reactions of other people been like? What have the reactions of people at school/teachers/other family been like? How does that make you feel? What do you think about that?

**5. How have things been in your family since your son/daughter had psoriasis/changes in their skin (use parent’s label for the skin condition)?**

Prompts: Has your psoriasis changed anything in your family? How have other people in your family been about your skin condition (go through each i.e. mum, dad, siblings)? Prompt – what sorts of things have been said/done in connection with your skin condition?

*Thanks & Debrief:* How have you found it talking with me? Do you want to add anything or alter anything you said previously? Do you have any questions? Is there anything that you’re worried about from our conversation? Is there anything you think I should ask other parents? Are you still happy for this recording to be used in my study?
